# Effect of Phenolic Compounds and Terpenes on the Flavour and Functionality of Plant-Based Foods

**DOI:** 10.3390/nu17213319

**Published:** 2025-10-22

**Authors:** Natalia Kurhaluk, Lyudmyla Buyun, Renata Kołodziejska, Piotr Kamiński, Halina Tkaczenko

**Affiliations:** 1 Institute of Biology, Pomeranian University in Słupsk, 76-200 Słupsk, Poland; halina.tkaczenko@upsl.edu.pl; 2 Department of Tropical and Subtropical Plants, M.M. Gryshko National Botanical Garden, National Academy of Sciences of Ukraine, 01103 Kyiv, Ukraine; orchids.lyuda@gmail.com; 3 Department of Medical Biology and Biochemistry, Collegium Medicum in Bydgoszcz, Nicholaus Copernicus University, M. Karłowicz St. 24, 85-092 Bydgoszcz, Poland; renatak@cm.umk.pl; 4 Department of Medical Biology and Biochemistry, Division of Ecology and Environmental Protection, Collegium Medicum in Bydgoszcz, Nicolaus Copernicus University in Toruń, M. Skłodowska-Curie St. 9, 85-094 Bydgoszcz, Poland; piotr.kaminski@cm.umk.pl; 5 Department of Biotechnology, Institute of Biological Sciences, Faculty of Biological Sciences, University of Zielona Góra, Prof. Z. Szafran St. 1, 65-516 Zielona Góra, Poland

**Keywords:** phytochemicals, volatile compounds, anticancer activities, inflammation, antioxidant properties, gut health, food processing, functional foods

## Abstract

**Background:** Phytochemicals play a crucial role in determining the sensory qualities and nutritional value of plant-based foods. They influence flavour perception by interacting with aroma, taste, and texture. Terpenes, phenolic compounds, and flavonoids are particularly important as they contribute to the characteristic sensory profiles of foods while offering antioxidant, anti-inflammatory and anticancer properties that support the prevention of diet-related chronic diseases. **Methods:** A systematic literature search was conducted in PubMed, Web of Science, Scopus, and EMBASE, complemented by Google Scholar. The search focused on peer-reviewed articles, reviews, and meta-analyses published within the last two decades, prioritising studies on phytochemicals, their biosynthesis, the molecular mechanisms of flavour formation, and their functional properties in plant-based foods. Keywords included ‘phytochemicals’, ‘flavour development’, ‘flavonoids’, ‘terpenes’, ‘phenolics’, ‘plant foods’, ‘molecular pathways’, and ‘food processing’. Relevant studies providing mechanistic insights were selected. **Results:** Terpenes, phenolic compounds, and flavonoids modulate sensory attributes by interacting with taste and olfactory receptors, and they contribute to antioxidant and anti-inflammatory mechanisms. Food processing influences the stability, bioavailability, and efficacy of these compounds, thereby affecting flavour and health-promoting potential. Modern analytical techniques enable the detailed characterisation of these compounds and their sensory and functional roles. **Conclusions:** By integrating insights from sensory science and nutrition, this review emphasises the dual importance of phytochemicals in enhancing consumer acceptance and promoting health. Understanding their mechanisms and how they respond to processing can inform the development of plant-based foods that are enjoyable and nutritious.

## 1. Introduction

Food and nutrition security are two major challenges faced by the growing global population, which is estimated to reach 9.7 billion by 2050 [1]. A balanced diet—rich in plant-based foods, high in dietary fibre, low in meat, and minimal in ultra-processed foods—has consistently been linked to improved metabolic health, enhanced immune function, greater stress resilience, and a reduced risk of genetic mutations, all of which are key factors in healthy aging [2].

Phytochemicals play a crucial role in shaping the flavour profile and functional properties of plant-based foods, as these bioactive compounds contribute to a wide range of sensory attributes, including bitterness, astringency, sweetness, and aroma. They are, therefore, integral to food quality and consumer acceptance [3,4]. In addition to their influence on organoleptic characteristics, phytochemicals have significant health-promoting properties, including antioxidant, anti-inflammatory, and antimicrobial activities [5]. As the demand for sustainable and health-conscious dietary choices continues to grow, a comprehensive understanding of the role of phytochemicals in flavour development remains a critical area of investigation within food science and nutrition research [6,7].

The growing preference for natural and functional foods reflects a fundamental shift in consumer behaviour. The shift toward plant-based diets has driven growing interest in foods that not only offer enhanced sensory appeal but also provide functional benefits [8,9]. Consumers are increasingly favouring minimally processed products without synthetic additives, underscoring the importance of naturally occurring phytochemicals as key modulators of taste and bioactivity [10]. In addition, the growing recognition of the relationship between diet and health has fuelled interest in functional foods, where phytochemicals serve a dual function—enhancing flavour while exerting physiological effects [5,11]. Their role as natural antioxidants and anti-inflammatory agents aligns with dietary strategies designed to reduce the risk of chronic diseases, including cardiovascular disorders, neurodegenerative conditions, and metabolic syndromes [12,13]. This underscores their broader importance within contemporary food systems and highlights their potential to improve both food quality and public health outcomes [14,15].

The molecular mechanisms underlying flavour development in plant-based foods remain incompletely understood and require further investigation. The flavour profile of these foods arises from complex biochemical interactions involving the biosynthesis, degradation, and conversion of phytochemicals during plant growth, storage, and processing [16]. For example, flavonoids contribute to bitterness and astringency through their interactions with taste receptors and salivary proteins [17,18], while terpenes impart distinct aromatic characteristics due to their volatile nature [19,20]. In addition, phenolic compounds influence not only taste perception but also oxidative stability, thereby affecting both shelf life and nutritional quality [21,22]. As the phytochemical composition is inherently variable, depending on plant species, cultivation conditions, and post-harvest treatments, extensive studies are required to optimise flavour profiles and ensure consistency in plant-based foods. Elucidation of these molecular pathways can inform food formulation strategies aimed at improving sensory attributes and consumer acceptance of plant-based alternatives [23].

The growing global demand for sustainably produced high-quality chocolate with unique flavour characteristics continues to drive interest in cocoa and chocolate research [24,25,26]. As chocolate production increasingly prioritises the preservation of cocoa’s intrinsic flavour attributes, research has focused on minimally processed approaches and the impact of fermentation on flavour development [27]. This field of research highlights the intersection of food science and production technology, demonstrating how advancements in processing methods can enhance product quality, reflect regional variability, and meet the preferences of health-conscious consumers seeking minimally processed authentic food experiences.

A deeper understanding of the role of phytochemicals in flavour development is critical to driving innovation in plant-based food science. Research in this area not only elucidates the biochemical pathways that govern flavour formation, but also facilitates the development of strategies to optimise sensory properties while preserving health benefits [11]. The application of advanced analytical techniques, including metabolomics and sensory evaluation, further enhances the understanding of phytochemical interactions within food matrices and their impact on human taste perception [28]. In particular, a study conducted by Bickel Haase et al. (2021) [25] highlights the importance of cocoa liquor (a by-product of cocoa bean processing) as a valuable ingredient with multiple culinary applications, contributing to both sustainability and the economic well-being of cocoa farmers. Their analysis of aroma compounds in cocoa liquor from different geographical regions revealed considerable variability in flavour profiles [25]. For example, *Vietnamese cocoa* pulp had the highest number of aroma-active compounds, indicating a complex and rich flavour profile, while Cameroonian pulp had the lowest number, suggesting a more muted flavour. Specific compounds, such as trans-4,5-epoxy-(E)-decenal, 2- and 3-methylbutanoic acid, and linalool, which had high flavour dilution (FD) factors, were identified as key contributors to the fruity and floral notes characteristic of cocoa pulp. These findings underscore the importance of a deeper understanding of regional flavour differences to support the development of innovative food products [25,29]. In addition, research into food processing technologies that preserve or enhance phytochemical content will be instrumental in creating plant-based products that are both flavourful and nutritionally beneficial [6,30]. This review aims to provide a comprehensive overview of the molecular mechanisms by which phytochemicals influence flavour, highlighting their potential applications in food science and human health.

This review examines the roles of terpenes, phenolic compaunds, and flavonoids in determining the sensory and functional properties of plant-based foods. It analyses the molecular pathways that regulate phytochemical biosynthesis and the interactions between these compounds and taste and olfactory receptors. It also evaluates their contribution to antioxidant and anti-inflammatory mechanisms. Additionally, it evaluates the impact of food processing on the stability and bioavailability of these compounds, offering strategies to preserve flavour and maximise health benefits in plant-based diets. By integrating the latest research, this review aims to help food scientists, nutritionists and the food industry design plant-based foods that combine sensory quality with nutritional value.

## 2. Methods and Scope of the Review

A systematic literature search was conducted in PubMed, Web of Science, Scopus and EMBASE, and was supplemented with a Google Scholar search to capture additional references. These sources were selected due to their extensive coverage of the biomedical sciences, food sciences, and phytochemistry. The search focused on peer-reviewed articles, reviews and meta-analyses published within the last twenty years. Studies addressing phytochemicals, their biosynthesis, the molecular pathways involved in flavour formation and their functional properties in plant-based foods were prioritised. A predefined combination of keywords was applied, including “phytochemicals”, “volatile compounds”, “flavour development”, “flavonoids”, “terpenes”, “phenolics”, “plant foods”, “molecular pathways”, and “food processing’. Abstracts were screened for relevance, and only those providing mechanistic insights into phytochemical-mediated flavour formation and biological activity were included. This review critically examines the role of key phytochemicals, including terpenes, phenolic compounds, and flavonoids in shaping the sensory and functional properties of plant foods. It offers an in-depth analysis of the molecular pathways regulating phytochemical biosynthesis, their interactions with taste and olfactory receptors, and their contributions to antioxidant and anti-inflammatory mechanisms. Additionally, the review explores the impact of food processing techniques on the stability and bioavailability of these compounds, offering insights into strategies for optimising both taste and health benefits in plant-based diets. By synthesising current scientific knowledge, this work provides valuable insights for food scientists, nutritionists, and the food industry, thereby contributing to the advancement of plant-based food innovation.

## 3. Molecular and Physiological Mechanisms of Flavour Perception in Plant-Based Foods

Flavour perception is a complex neurophysiological process that integrates sensory inputs from the gustatory and olfactory systems, ultimately enabling the recognition of distinct flavour profiles [31]. This multisensory process begins when volatile organic compounds (VOCs) released from food interact with olfactory receptors in the nasal cavity, while non-volatile compounds, such as amino acids, sugars, and organic acids, bind to specific taste receptors on the tongue. These signals are then processed in the central nervous system, leading to the conscious perception of taste [32].

**Figure 1:**
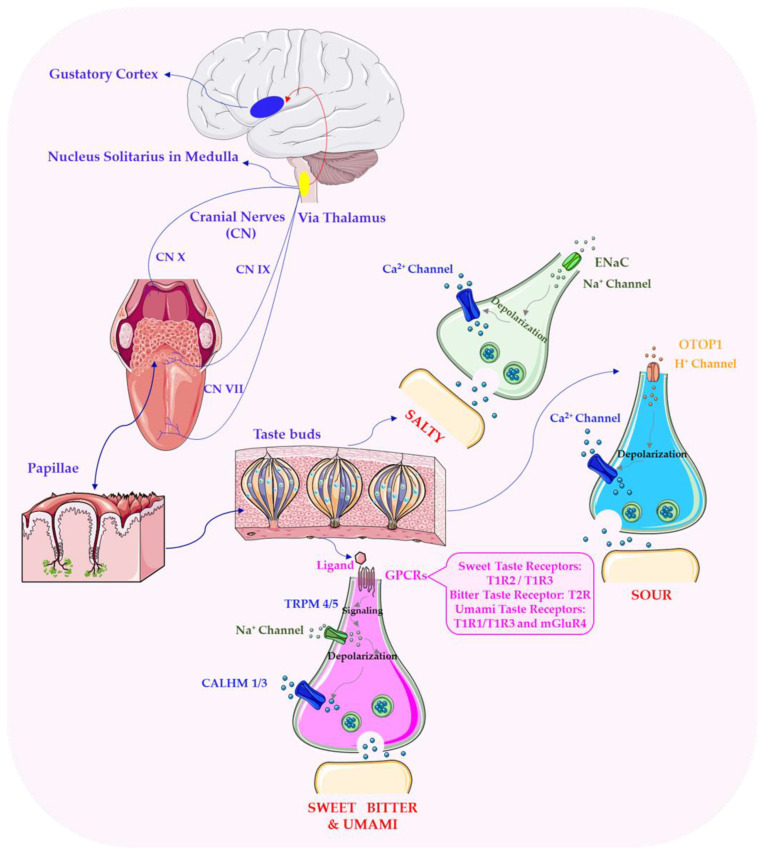
Neural pathway of taste perception. Abbreviations: CALHM 1/3—Calcium homeostasis modulator 1/3; CN—Cranial Nerves; ENaC—Epithelial sodium channel; GPCRs—family of G-protein–coupled receptors (mGluR4—metabotropic glutamate receptor; T1R1—taste receptor type 1 member 1; T1R2—taste receptor type 1 member 2; T1R3—taste receptor type 1 member 3; T2R—taste receptor type 2); OTOP1—Otopetrin 1; TRPM 4/5—Ca^2+^-activated monovalent cation channels. Image provided by Servier Medical Art (https://smart.servier.com/), licensed under CC BY 4.0 (https://creativecommons.org/licenses/by/4.0/, accessed on 1 May 2025).

At a physiological level, flavour perception is governed by two primary sensory systems: the gustatory system, responsible for detecting the five basic tastes—sweet, sour, bitter, salty, and umami—and the olfactory system, which detects volatile aroma compounds [33,34]. The olfactory system operates through two primary pathways: orthonasal olfaction, which occurs when volatile compounds are inhaled through the nostrils, and retronasal olfaction, which takes place when these compounds are released in the oral cavity during chewing and transported through the nasopharynx to the olfactory epithelium. This retronasal pathway plays a crucial role in taste perception, as it integrates with gustatory inputs to produce the characteristic sensory experience of food [35,36]. Neural pathway of taste perception shown in Figure 1. Figure 1 illustrates the transmission of taste signals—from taste receptors located on the tongue and in the oral cavity, through the cranial nerves (VII, IX, X), to the nucleus of the solitary tract in the medulla, then to the thalamus, and finally to the gustatory cortex, where taste stimuli are integrated with other sensory modalities.

At the molecular level, taste receptors belong to the G-protein-coupled receptor (GPCR) superfamily, which mediates the detection of specific chemical compounds [37]. For example, sweetness is primarily detected by the T1R2/T1R3 receptor complex, which binds to mono- and disaccharides (e.g., glucose, fructose, sucrose) as well as certain artificial sweeteners and sugar alcohols [38,39]. Bitter taste perception is mediated by the T2R receptor family, which recognises a wide range of structurally diverse bitter compounds, including alkaloids, polyphenols, and terpenoids [40,41]. The detection of sourness is associated with proton-sensing ion channels that respond to acidic compounds, such as citric acid and acetic acid, while the umami taste is triggered by the binding of L-glutamate and other amino acids to the T1R1/T1R3 receptor complex [42,43]. Saltiness, on the other hand, is primarily mediated by epithelial sodium channels (ENaCs) that detect sodium ions and other mineral salts [43,44].

The olfactory system is responsible for detecting volatile compounds, such as aldehydes, alcohols, esters, terpenes, and sulphur-containing molecules, many of which contribute significantly to the aromatic complexity of plant foods [45]. These compounds are recognised by a large repertoire of olfactory receptors located in the nasal epithelium, with each receptor exhibiting specificity for a particular subset of molecules. The interaction between taste and aroma compounds creates a synergistic effect that enhances the depth and complexity of flavour perception [46,47].

Beyond the molecular interactions at the receptor level, the integration of gustatory and olfactory signals occurs in higher-order brain regions, including the gustatory cortex, olfactory cortex, and orbitofrontal cortex [48,49]. These regions are responsible for processing and synthesising sensory input to facilitate conscious perception of taste [49]. In addition, multisensory integration mechanisms play a crucial role in modulating flavour perception, as such factors as texture, temperature, and previous sensory experiences contribute to an individual’s interpretation of food flavour [50,51].

**Figure 2:**
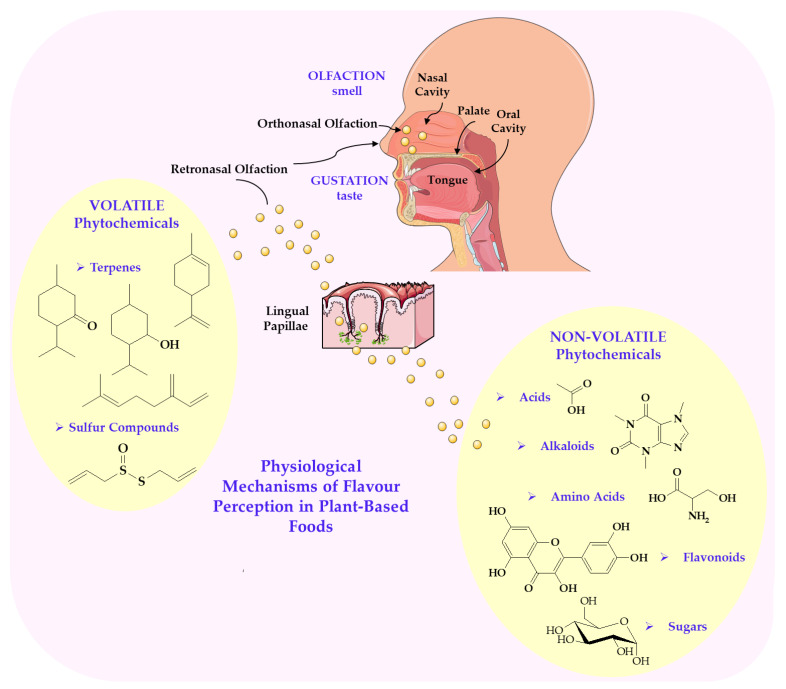
Mechanism of physiological perception of taste of substances of plant origin. Image provided by Servier Medical Art (https://smart.servier.com/), licensed under CC BY 4.0 (https://creativecommons.org/licenses/by/4.0/, accessed on 1 May 2025).

In plant-based foods, the composition of flavour-determining molecules, including VOCs, amino acids, lipids, and secondary metabolites, varies widely depending on the species, plant cultivar, ripening stage, cultivation practices, and post-harvest processing [45,52]. For example, flavonoids and phenolic acids contribute to bitterness and astringency, while terpenes are responsible for distinct aromatic notes [53]. Lipid oxidation products, such as aldehydes and ketones, play an important role in creating the characteristic flavour of vegetable fats and oils [54,55]. The interactions of these compounds with sensory receptors are fundamental in defining the organoleptic properties of plant-based foods, as illustrated in Figure 2.

A deeper understanding of the molecular and physiological mechanisms underlying flavour perception is essential for optimising the sensory quality of plant-based foods. Advances in flavour chemistry, sensory science and food processing technologies have significant potential to improve the acceptability and consumer preference for plant-based alternatives while maintaining their nutritional and functional benefits [56].

Taste perception is a complex neurophysiological process involving the integration of gustatory, olfactory, and somatosensory signals in the central nervous system [57]. Taste perception is a key element in the interaction between organisms and their environment, as it provides crucial information about the quality and nutritional value of consumed food and allows humans to detect potential dietary hazards [58].

Taste perception begins when chemical stimuli interact with specialised taste receptors located on the tongue and in the oral cavity. These receptors, which belong to the G-protein-coupled receptor (GPCR) and ion channel families, detect the five primary taste modalities: sweet, sour, bitter, salty, and umami [37,42]. When activated, afferent signals from these receptors are transmitted via the cranial nerves (the facial nerve [cranial nerve VII], the glossopharyngeal nerve [cranial nerve IX], and the vagus nerve [cranial nerve X]) to the gustatory nucleus in the nucleus tractus solitarius of the medulla oblongata. From there, signals are relayed to the thalamus, which serves as a critical processing hub, before being projected to the gustatory cortex in the parietal lobe. The gustatory cortex not only deciphers basic taste modalities but also integrates the taste input with other sensory modalities, such as texture and mouthfeel, contributing to the holistic perception of flavour [42,57].

At the same time, olfactory perception is triggered when VOCs released from food bind to olfactory receptors embedded in the olfactory epithelium of the nasal cavity [59]. These receptors, which belong to the largest family of GPCRs in the human genome, detect a wide range of chemical structures and generate neural signals that are transmitted to the olfactory bulb [60]. The olfactory bulb performs primary signal processing before relaying the information to higher brain regions, including the piriform cortex, the amygdala, and the orbitofrontal cortex. The integration of olfactory signals in these regions enables the identification and interpretation of complex aroma profiles that are essential for overall taste perception [57,61].

A critical aspect of taste perception is the multisensory integration of gustatory and olfactory inputs that occurs in higher-order brain regions, particularly the insular cortex and the orbitofrontal cortex [62,63]. The insular cortex, a key centre for interoceptive awareness, plays a central role in merging taste perception with other sensory attributes, such as texture, temperature, and oral somatosensory feedback [64]. The orbitofrontal cortex, on the other hand, serves as a central hub for integrating olfactory, gustatory, and visual cues, facilitating the conscious perception of taste and contributing to the hedonic evaluation of food [65,66]. In addition, the anterior cingulate cortex and the prefrontal cortex are involved in the cognitive and emotional aspects of taste perception, including decision-making processes related to food preference and reward-driven eating behaviour [67,68].

Importantly, the limbic system, particularly the amygdala and the hippocampus, plays a fundamental role in linking flavour perception to emotional and memory-related processes [57,69]. The strong link between the olfactory system and limbic structures explains the well-documented phenomenon in which certain flavours can evoke vivid memories and emotional responses [70]. This link has significant implications for dietary behaviour, as taste preferences and aversions are often shaped by past experiences and conditioned responses [32].

In addition to taste and smell, other sensory modalities contribute to the overall perception of flavour. Temperature influences the activation of taste receptors; for example, the perception of sweetness is enhanced at warmer temperatures, while bitterness may be more pronounced at lower temperatures [71]. Similarly, food texture, perceived by mechanoreceptors in the oral cavity, affects the mouthfeel and palatability of a food product [72]. In addition, visual cues, including colour and presentation, have a significant impact on flavour expectations and perception, as evidenced by studies showing that changes in food colour can alter the perceived intensity of certain flavour attributes [73].

Taste perception is also subject to genetic, environmental, and cultural influences. Genetic polymorphisms in taste receptors, particularly within the TAS2R family responsible for bitter taste perception, contribute to individual differences in taste sensitivity and food preferences [74,75]. Environmental factors, including dietary exposures and early life feeding experiences, play a role in shaping taste perception over time [76,77]. In addition, cultural traditions and culinary practices influence taste preferences by modulating sensory expectations and habitual consumption patterns [78].

Among plant foods, chocolate serves as an exemplary model for studying flavour complexity due to its rich sensory profile and extensive biochemical transformations during processing. The research conducted by Ullrich et al. (2022) provides significant insights into the molecular mechanisms underlying chocolate flavour, with a particular focus on key sensory attributes, such as fruity, floral, and cocoa-like notes [79]. Using aroma extract dilution analysis, the study identified key odour compounds, including esters and aldehydes, contributing to the characteristic chocolate aroma. Acetic acid and fruity esters were found to enhance acidic and fruity flavour components, while sulphur-containing compounds and flavonoids, such as (-)-epicatechin and procyanidins, were associated with roasted and floral attributes. These findings highlight the importance of molecular profiling in flavour optimisation and provide a novel framework for refining the sensory properties of chocolate [79].

## 4. Functional Attributes in Food as Quality Indicators

There is a growing global trend in the search for functional attributes in food as quality indicators. In the case of cocoa beans, the final qualitative and quantitative content of polyphenols and methylxanthines might be considered as part of the quality indicators affecting the final price of the beans [80]. A critical factor influencing chocolate flavour is the cocoa bean fermentation process, which alters their biochemical composition and impacts the final product [80,81]. A study reported by Lima et al. (2011) highlights the crucial role of microbial consortia, particularly *Bacillus* spp., in shaping cocoa fermentation outcomes [82]. While the microbial sequence of fermentation varies geographically, key metabolic by-products, including organic acids, alcohols, and volatiles, directly influence flavour development. Further research integrating metagenomic and metabolomic approaches is needed to elucidate the interactions between microbial diversity and flavour precursors, thereby enabling targeted interventions to standardise and enhance cocoa quality. Similarly, Seyfried and Granvogl (2019) and Schlüter et al. (2022) emphasise that such variables as pod ripeness, fermentation duration, and postharvest processing significantly influence cocoa aroma and flavour [83,84].

Recent research carried out by Ac-Pangan et al. (2023) provides further evidence of the crucial role of thermal processing in flavour development [85]. Their study shows that roasting enhances the release of key flavour compounds, such as dimethyltrisulphide and 2-ethyl-3,5-dimethylpyrazine, which contribute to the characteristic roasted chocolate flavour. However, the study also shows that minimal thermal processing techniques help preserve certain fermentation-derived aroma compounds, such as acetic acid, supporting the concept of ‘chocolate terroir’—a term referring to regional and process-related variations in flavour. These results suggest that adjusting of roasting parameters may help preserve or enhance of specific sensory attributes, providing a means to tailor chocolate products to consumer preferences [85].

## 5. Identification and Quantification of Flavour Compounds in Plant Foods

The sensory attributes of foods, particularly flavour and aroma, are determined by the intricate interplay of volatile and non-volatile compounds that originate from the raw materials used, the biochemical transformations that occur, and the processing techniques employed. These factors all influence consumer perception and product acceptability [4,86]. Accurate identification and quantification of these compounds requires advanced analytical methods combining high-resolution chemical separation, mass spectrometry and sensory evaluation [87].

Among the various instrumental approaches, gas chromatography–mass spectrometry (GC-MS) and liquid chromatography–mass spectrometry (LC-MS/MS) are widely used due to their superior separation efficiency and detection sensitivity [88,89]. GC-MS separates volatile organic compounds (VOCs) based on volatility and polarity, and then identifies them by analysing their mass spectra [90,91]. This technique enables the detection of trace levels and the comprehensive profiling of flavour-active volatiles, providing insights into the formation, degradation and evolution of aroma compounds during food processing and storage [92,93]. In contrast, proton transfer reaction mass spectrometry (PTR-MS) provides real-time monitoring of volatile compound dynamics without requiring chromatographic separation. This enables the rapid analysis of headspace volatiles in complex matrices, such as fermented foods, coffee and chocolate [94,95,96,97,98]. Nuclear magnetic resonance (NMR) spectroscopy also contributes by providing detailed structural and quantitative information on non-volatile metabolites, such as polyphenols, alkaloids and flavonoids, which influence taste and mouthfeel [99,100,101].

However, instrumental analyses alone are insufficient for fully correlating chemical composition with human sensory experience. Sensory evaluation remains indispensable in flavour research and requires trained sensory panels that can detect subtle differences in taste and aroma [102]. Integrating gas chromatography-olfactometry (GC-O), which couples instrumental detection with sensory assessment, enables the identification of key odour-active compounds with low perception thresholds that directly influence consumer response [103,104]. Similarly, taste-activity-guided fractionation using HPLC-MS/MS enables the isolation and characterisation of taste-active molecules, such as amino acids, peptides and polyphenols, which are linked to umami, bitterness or astringency [105,106]. By combining molecular data with sensory evaluation, researchers can establish meaningful correlations between chemical profiles and organoleptic attributes [107].

These methods have been applied to various food systems, including cocoa, coffee and *Cannabis sativa* L. inflorescences. GC-MS and LC-MS analyses have revealed dynamic changes in phenolic compounds, flavonoids and terpenes across different processing or developmental stages [108,109,110,111,112]. Such studies emphasise the importance of advanced analytics in optimising ingredient quality, harvest timing and formulation strategies. Insights from flavour research in chocolate and other plant-based products support the development of improved sensory balance in products by addressing issues such as bitterness, unpleasant aromas, and inadequate umami perception. Techniques such as enzymatic treatment, fermentation, and encapsulation are promising approaches for modulating plant-based flavours and enhancing consumer acceptance [113,114,115].

Advanced analytical techniques play a vital role in ensuring food safety by detecting and quantifying harmful contaminants such as mycotoxins [116,117]. Studies by Mischler et al. (2024) have demonstrated the potential of *Bacillus licheniformis* for the biological detoxification of mycotoxins such as enniatin B and deoxynivalenol in cereals [118]. These studies have also highlighted the complementary roles of GC-MS and HPLC-MS/MS in monitoring detoxification efficiency. The integration of microbial detoxification strategies with analytical validation contributes to the development of sustainable, low-additive food production systems [119,120].

Together, the comprehensive characterisation of flavour and aroma compounds in plant foods necessitates a multidisciplinary approach that combines advanced analytical techniques with sensory science. Uniting methods such as GC-MS, GC-O, HPLC-MS/MS, PTR-MS and NMR spectroscopy enables researchers to connect molecular composition to human sensory perception, ensuring product safety and sensory excellence. Furthermore, using biotechnology and precision fermentation to produce or improve flavour-active compounds is an emerging area of sustainable food innovation [56,121]. These integrative strategies improve the quality and acceptability of plant-based foods, supporting dietary transitions towards healthier and more environmentally responsible food systems [6,121].

## 6. Phytochemical Classes and Molecular Interactions Underlying Flavour Perception in Plant-Based Foods

The complex relationship between phytochemicals and human sensory perception is fundamental to the wide range of flavours and aromas found in plant-based foods. These bioactive compounds, primarily terpenes, phenolic compounds, and flavonoids, modulate taste and aroma by interacting with gustatory and olfactory receptors, salivary proteins and the enzymatic systems involved in flavour formation and modification [4,122,123]. Beyond their sensory functions, phytochemicals also enhance the nutritional value, oxidative stability and overall acceptability of foods [124,125].

Phenolic acids and tannins, which are derived from the shikimate pathway, play a dual role in determining the sensory and functional properties of plant-based foods [126]. They impart bitterness and astringency by interacting with salivary proteins, causing proline-rich proteins to precipitate and resulting in the sensation of oral dryness [127,128,129]. Furthermore, phenolic compounds exhibit strong antioxidant activity, stabilising volatile compounds by inhibiting their oxidative degradation and helping to maintain flavour integrity during storage [53,130,131]. Their contribution extends beyond sensory effects to enhance the nutritional and preservative properties of plant-based foods [132].

Flavonoids are a structurally diverse class of polyphenolic compounds synthesised via the phenylpropanoid pathway. This pathway is initiated by the conversion of phenylalanine to cinnamic acid via the enzyme phenylalanine ammonia-lyase (PAL) [133]. This pathway produces subclasses such as anthocyanins, flavonols, flavanones and flavones, which contribute different sensory and visual properties [134,135]. Flavonoids primarily influence flavour by inducing bitterness and astringency through binding to bitter taste receptors (TAS2Rs) and interacting with salivary proteins, thereby altering mouthfeel [18,136]. Compounds such as quercetin, catechins and kaempferol also modulate gustatory G-protein-coupled receptors (GPCRs), thereby affecting taste signal transduction downstream and overall sensory perception [137,138]. Furthermore, their pigmentation properties indirectly influence flavour perception by providing visual cues that shape consumer expectations [139].

**Figure 3:**
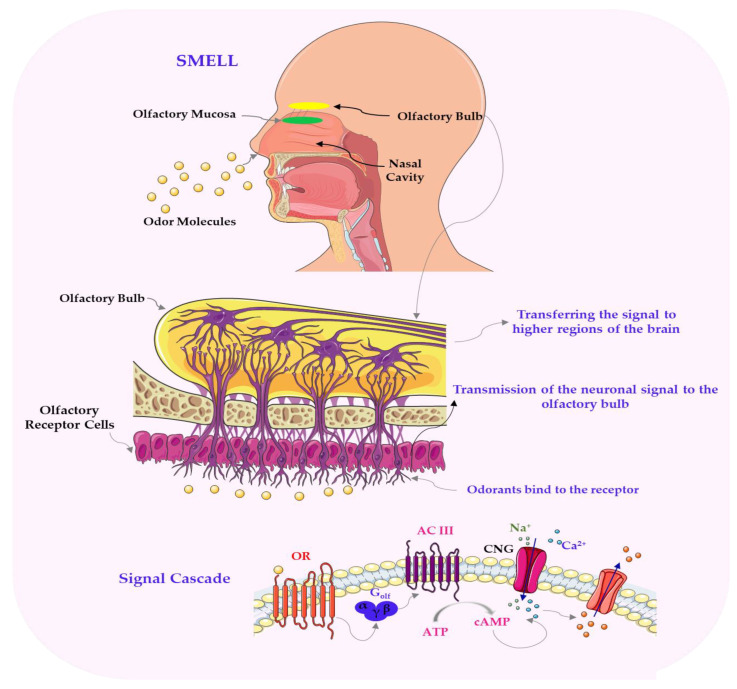
Signaling cascade triggered by odorants. Abbreviations: AC III—Adenylyl cyclase III; ATP—Adenozyno-5′-trifosforan; cAMP—Cyclic adenosine monophosphate; CNG—Cyclic nucleotide-gated; G_olf_—specific G protein; OR—Olfactory receptor. Image provided by Servier Medical Art (https://smart.servier.com/), licensed under CC BY 4.0 (https://creativecommons.org/licenses/by/4.0/, accessed on 1 May 2025).

Terpenes are a major class of volatile organic compounds that are responsible for the characteristic aromas of herbs, citrus fruits and spices [140,141]. They are synthesised via the mevalonate (MVA) and methylerythritol phosphate (MEP) pathways and encompass monoterpenes, sesquiterpenes, and diterpenes, which interact with olfactory receptors (ORs) in the nasal epithelium [142,143,144]. Their volatility enables immediate sensory detection, resulting in citrus, woody, or floral notes depending on their molecular structure: limonene imparts a fresh citrus scent, myrcene evokes earthy tones, and linalool provides floral and spicy nuances [20,145,146]. In addition to their olfactory roles, terpenes exhibit antimicrobial and antioxidant properties that improve product safety and extend shelf life [21,147]. Mechanisms and key volatile compounds in the flavour profiles of plant-based foods, e.g., terpenes like limonene and myrcene, interact with olfactory receptors to produce distinctive aromas that contribute to citrus, earthy, and floral notes in plant-based products, as shown in Figure 3. As illustrated in Figure 3, the process of signal transduction initiated by odorant molecules is initiated by the activation of olfactory receptors (ORs). These receptors stimulate a specific G protein called Golf, which in turn activates type III adenylyl cyclase to synthesize cAMP from ATP. The resulting cAMP opens CNG ion channels, allowing the influx of Na^+^ and Ca^2+^ ions into the cell. The rise in intracellular Ca^2+^ concentration subsequently activates calcium-dependent chloride (Cl^−^) channels, leading to the efflux of Cl^−^ ions. This further depolarizes the cell membrane. Ultimately, this sequence of events results in the generation of an action potential, which is transmitted along the olfactory neuron to higher brain centers responsible for odor perception.

The biosynthesis of phytochemicals is tightly regulated by enzymatic activity, gene expression and environmental stimuli. Flavonoids, terpenes and phenolic acids originate from different yet interconnected pathways—the phenylpropanoid, MVA/MEP and shikimate pathways—whose flux is modulated by stress signals, the developmental stage and metabolic cross-talk [148,149]. External factors such as light intensity, temperature variation and soil composition can also influence the quantitative and qualitative composition of phytochemicals, contributing to variability in flavour profiles among plant species and cultivation systems [150,151]. Understanding these regulatory mechanisms is essential for optimising agricultural, post-harvest and processing practices that enhance the sensory and nutritional value of plant-based foods.

Thanks to advances in analytical chemistry, we now have a much better understanding of the role of phytochemicals in flavour perception. Techniques such as GC-MS, HPLC-MS/MS, and NMR spectroscopy enable detailed profiling of both volatile and non-volatile compounds [87]. They allow us to elucidate the complex interactions between phytochemicals, sensory receptors, and metabolic processes, offering new insights into how to optimise flavour and functionality in food design [152]. Integrating molecular, sensory, and analytical approaches enables researchers to harness phytochemical diversity more effectively, enhancing the sensory appeal and health-promoting properties of plant-based foods [124,125].

### 6.1. Terpenes in Plant-Based Foods: Aromatic Complexity and Functional Properties

Terpenes are a diverse class of naturally occurring organic compounds that play a crucial role in shaping the flavour profiles of plant-based foods and beverages [20]. These volatile molecules are responsible for the characteristic aromas that define the sensory experience of fruits, vegetables, herbs, spices, and essential oils [153]. For example, citrus fruits such as oranges, lemons and grapefruits owe their refreshing flavour primarily to limonene [154,155], while mangoes, hops and lemongrass are rich in myrcene [156]. The distinctive notes of apples, peppermint, rosemary and cloves are derived from various terpenes, including menthol, pinene and eugenol [157,158,159].

Depending on their molecular structure and concentration, terpenes such as limonene, pinene, myrcene, linalool and terpinene contribute distinct aromatic qualities—citrusy, pine-like, earthy or floral [160,161,162]. Other compounds, such as caryophyllene, geraniol, humulene and cineole, enrich the sensory experience with spicy, sweet, woody and menthol-like notes [143,163,164,165]. These terpenes enhance flavour and possess antioxidant, anti-inflammatory and antimicrobial properties, thereby contributing to the nutritional and therapeutic value of plant-based products [19,141,144].

Terpenes are synthesised via the terpenoid biosynthetic pathway involving enzymes such as terpene synthases, which convert precursors such as geranyl pyrophosphate (GPP) into specific terpenes [166]. The MEP and MVA pathways regulate this process [166]. Once formed, terpenes interact with olfactory receptors in the nasal cavity, triggering neural responses that result in aroma perception [144]. They also modulate taste receptors, influencing sweetness or bitterness [74].

**Figure 4:**
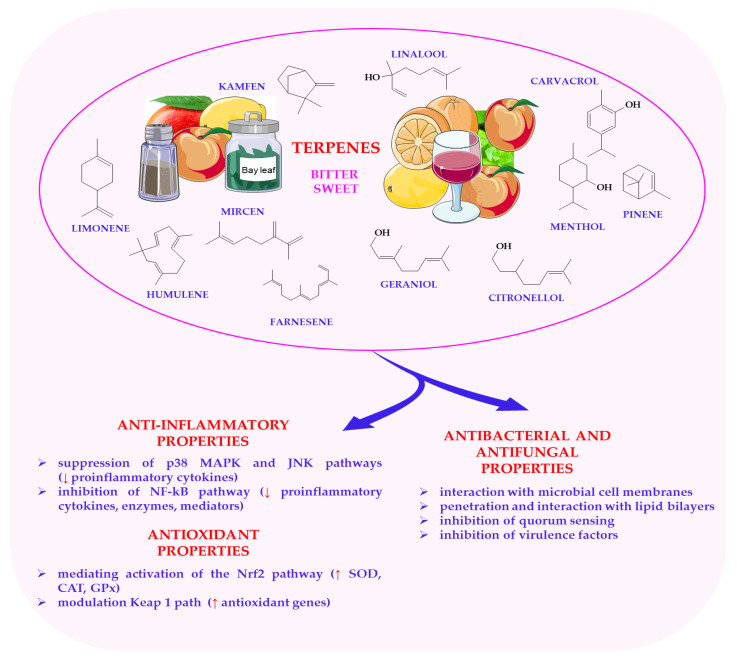
Molecular mechanisms of terpene action. Abbreviations: CAT—Catalase; GPx—Glutathione peroxidase; JNK—Janus kinase; p38 MAPK—p38 mitogen-activated protein kinase; NF-кB—Nuclear factor kappa-light-chain-enhancer of activated B cells; Nfr2—Nuclear factor erythroid 2-like 2; SOD—Superoxide dismutase. The red arrows indicate a decrease (↓) or increase (↑) in the respective parameter. Image provided by Servier Medical Art (https://smart.servier.com/), licensed under CC BY 4.0 (https://creativecommons.org/licenses/by/4.0/, accessed on 1 May 2025).

The release of volatile terpenes, such as β-caryophyllene and linalool, during food processing (e.g., heating, crushing, or fermentation) further alters the aroma profile of foods, introducing fresh, floral, citrus, or spicy notes that directly affect the overall flavour experience [167]. Terpenes also exhibit synergistic interactions with other bioactive compounds, including flavonoids, phenolics, and lipids, which can enhance or modify flavour perception [20,21]. It is important to note that each terpene has a different odour threshold, with certain compounds, such as limonene and pinene, having very low thresholds, meaning even small amounts can significantly affect the aroma and flavour of plant-based foods [168]. The molecular mechanisms by which terpenes exert their antioxidant, anti-inflammatory, and antimicrobial properties are shown in Figure 4. This figure shows that terpenes act through various mechanisms, including the modulation of signalling pathways, the activation of detoxifying enzymes and the regulation of gene expression related to inflammation and oxidative stress. Their antioxidant properties stem from the ability to scavenge free radicals, helping to protect cells from oxidative damage. Terpenes also exhibit anti-inflammatory effects by inhibiting the production of pro-inflammatory cytokines and modulating transcription factors such as NF-κB. Additionally, many terpenes have antimicrobial activity, affecting microbial cell membranes and disrupting their metabolic processes.

During food processing, such as heating, crushing or fermentation, volatile terpenes such as β-caryophyllene and linalool are released. This alters the aroma profile and enhances flavour complexity [167]. Their interactions with flavonoids, phenolic compounds and lipids further modulate sensory perception [21]. These interactions not only enhance the sensory experience, but may also influence the bioavailability of other bioactive compounds, opening up new avenues of research into food functionality.

Recent studies have elucidated the molecular mechanisms underlying terpene-induced sensory effects. For example, limonene, linalool and pinene activate olfactory receptors via GPCR pathways, leading to the generation of secondary messengers such as cAMP and Ca^2+^. These depolarise sensory neurons and transmit aroma signals to the brain [169,170]. Additionally, terpenes such as linalool interact with gustatory receptors to modulate taste perception [171]. Their interactions with lipids during volatilisation enhance the release of other flavour compounds [20]. From a food technology perspective, these mechanisms could be used to design products with optimised sensory and health-promoting properties. However, our current understanding of odour thresholds and their interaction with other ingredients requires further experimental validation.

In conclusion, terpenes such as limonene, myrcene and pinene play a crucial role in the aromatic complexity and health-promoting properties of plant-based foods. Their biosynthesis, receptor interactions, and synergistic effects with other bioactive compounds highlight their significance in the fields of food science and nutrition [147,172]. From a functional foods perspective, terpenes should be considered integral elements of strategies to improve the sensory quality and health value of plant products, not just aromatic ingredients.

### 6.2. Phenolic Compounds in Plant Foods

Phenolic compounds constitute a broad class of natural bioactive molecules that play a pivotal role in determining the flavour, colour and sensory attributes of plant-based foods and beverages [172,173]. This group includes phenolic acids, flavonoids and tannins, which give fruits, vegetables, herbs, spices and processed foods their characteristic bitterness, astringency and flavour complexity [124,174]. For example, the astringency and mild bitterness of apples and pears are determined by chlorogenic acid [175], while berries acquire their tart and earthy notes from ellagic acid and tannins [176,177]. In beverages such as tea, coffee, and red wine, tannins, catechins, and resveratrol shape the sensory profile and health-promoting potential [178,179].

Herbs and spices, including cloves, cinnamon and oregano, have diverse flavour profiles due to their high phenolic content, particularly eugenol and rosmarinic acid [180,181]. Phenolic acids also define the robust flavours of darker honeys [182,183] and dark chocolate [184]. While the associations between specific phenolic compounds and flavour traits are well documented, there is still much to learn about how processing and matrix effects alter sensory outcomes in real food systems.

Beyond their sensory contributions, phenolic compounds exhibit strong antioxidant, anti-inflammatory and anticancer properties [185,186]. They scavenge free radicals, modulate redox homeostasis and prevent oxidative damage to lipids, proteins and DNA [187,188]. These effects are partly mediated by the activation of the Nrf2 pathway, which enhances the expression of endogenous antioxidant enzymes [189,190] COX-2, TNF-α and IL-6, thereby modulating inflammation and protecting tissues from chronic damage [191,192].

Phenolic compounds also demonstrate metabolic and enzymatic regulatory functions, including the inhibition of α-glucosidase and α-amylase, which support postprandial glucose control [193,194]. Through AMPK activation, they enhance insulin sensitivity and energy metabolism [195]. However, much of the current evidence is based on in vitro or animal models, and clinical validation of these effects in humans remains limited and inconsistent.

The anticancer properties of phenolic compounds involve the modulation of cell cycle regulators (p21 and p27) and tumour suppressor genes, such as p53, resulting in cell cycle arrest, the induction of apoptosis, and the inhibition of angiogenesis via the downregulation of VEGF [196,197,198]. Numerous studies confirm their role in reducing the incidence of colon, breast, liver and lung cancers [199,200,201,202].

**Figure 5:**
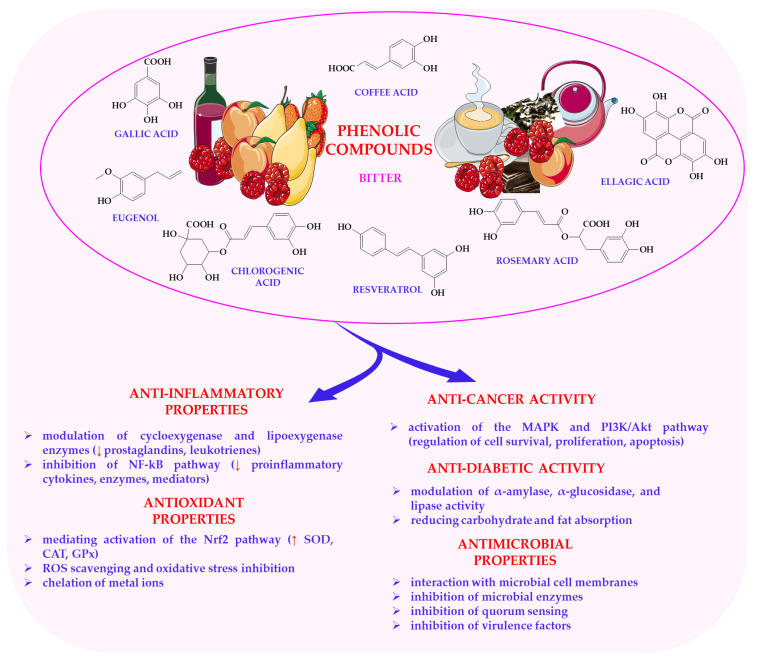
Molecular mechanisms of polyphenols’ action. Abbreviations: CAT—Catalase; GPx—Glutathione peroxidase; MAPK—Mitogen-activated protein kinase; NF-кB—Nuclear factor kappa-light-chain-enhancer of activated B cells; Nfr2—Nuclear factor erythroid 2-like 2; PI3K/Akt—Phosphatidylinositol 3-kinase/Protein kinase B; ROS—Reactive oxygen species; SOD—Superoxide dismutase. The red arrows indicate a decrease (↓) or increase (↑) in the respective parameter. Image provided by Servier Medical Art (https://smart.servier.com/), licensed under CC BY 4.0 (https://creativecommons.org/licenses/by/4.0/, accessed on 1 May 2025).

Recent evidence also highlights the neuroprotective effects of flavonoids such as quercetin and kaempferol. These compounds modulate the cholinergic and GABAergic systems, inhibit acetylcholinesterase and reduce neuronal excitability. This supports cognitive health [203,204,205]. Despite these promising mechanistic insights, translating them into dietary recommendations requires careful consideration of bioavailability, metabolism, and interindividual variability. The molecular mechanisms of phenolic compounds, including antioxidant, anti-inflammatory, enzyme inhibition and cell signalling modulation, provide a wide range of health benefits, as shown in Figure 5. This figure shows that polyphenols exhibit strong anti-inflammatory properties by inhibiting the expression of pro-inflammatory cytokines and enzymes, primarily by suppressing the NF-κB signalling pathway. They also inhibit the activity of key inflammatory enzymes such as cyclooxygenase and lipoxygenase. As potent antioxidants, polyphenols neutralize ROS and enhance the activity of endogenous antioxidant enzymes. Polyphenols demonstrate anticancer, antidiabetic, and antimicrobial properties. They modulate important cellular signaling pathways, such as MAPK and PI3K/Akt, which are involved in cell proliferation, apoptosis, and metabolic regulation. Moreover, they reduce the absorption of carbohydrates and lipids in the digestive tract, contributing to the maintenance of metabolic balance. Polyphenols interact with microbial cell membranes, disrupting their structure and function.

From a sensory perspective, tannins and flavonoids play a crucial role in flavour perception and mouthfeel [206,207]. Tannins interact with salivary proteins to produce the astringency typical of tea, wine and some fruits [128], while flavonoids such as quercetin and epicatechin are associated with bitterness and astringency [208,209]. These interactions influence taste receptor activation and the overall sensory balance of plant-based foods. Nevertheless, excessive astringency can limit consumer acceptance, which highlights the importance of optimising processing parameters and matrix composition in functional food development.

Dark chocolate is a particularly illustrative example, being rich in gallic, caffeic, and ferulic acids, as well as proanthocyanidins and chlorogenic acids, which define its complex bitterness, astringency, and aroma [184,210,211,212]. These same compounds also underpin its antioxidant capacity and potential cardiometabolic benefits [213].

In summary, phenolic compounds, particularly flavonoids and tannins, are multifunctional dietary components that contribute to the sensory quality and bioactive potential of plant-based foods. Their antioxidant, anti-inflammatory, anticancer, neuroprotective and metabolic regulatory properties highlight their importance in health promotion and chronic disease prevention [132,173,214]. Future studies should prioritise integrative models that combine sensory science, metabolomics and clinical trials, in order to bridge the gap between mechanistic understanding and practical dietary application.

### 6.3. Flavonoids in Health-Promoting Effects of Plant-Based Foods

Flavonoids are a large and structurally diverse class of polyphenolic compounds that are widely distributed in plant-based foods. They are recognised for their antioxidant, anti-inflammatory and multifaceted health-promoting properties [53,135,215]. Structurally, flavonoids share a C6–C3–C6 backbone composed of two aromatic rings linked by a three-carbon bridge [216]. The main subclasses are flavones, flavonols, flavanones, isoflavones, anthocyanins and flavan-3-ols [216,217]. Common dietary sources include quercetin, kaempferol, catechins and anthocyanins, which are found in abundance in fruits, vegetables, tea, red wine and dark chocolate [218]. Their structural diversity gives rise to a wide spectrum of biological and sensory activities, making them a major focus of research in nutrition and phytochemistry [219].

Flavonoids modulate key molecular pathways linked to inflammation, oxidative stress and cellular signalling [220,221,222]. Two of these, quercetin and catechins, have been extensively studied for their protective roles in cardiovascular, neurodegenerative and neoplastic diseases [223,224,225]. These effects are largely attributed to the inhibition of the NF-κB signalling cascade, which results in the downregulation of pro-inflammatory mediators such as TNF-α, IL-6, COX-2 and iNOS [226,227]. Concurrently, activation of the Nrf2 pathway induces antioxidant enzymes such as SOD, GPx and CAT, thereby protecting cells from oxidative damage [228].

Flavonoids also regulate the MAPK cascade, mitigating inflammatory responses by suppressing p38 MAPK and JNK signalling [229,230]. This molecular versatility contributes to their therapeutic potential in conditions such as arthritis [231], asthma [232,233] and inflammatory bowel disease [234]. However, despite robust in vitro and in vivo data, human clinical trials have been inconsistent in demonstrating dose-dependent effects, likely due to the poor bioavailability and rapid metabolism of flavonoids.

Flavonoids exert anticancer effects by modulating apoptosis and cell cycle control, which involves activating p53 and upregulating caspase cascades [235,236,237]. They suppress angiogenesis and inhibit metastasis, offering a promising addition to conventional cancer therapies [238]. Nonetheless, translating these preclinical findings into clinical outcomes requires addressing issues of dosage, synergistic interactions and metabolic conversion.

**Figure 6:**
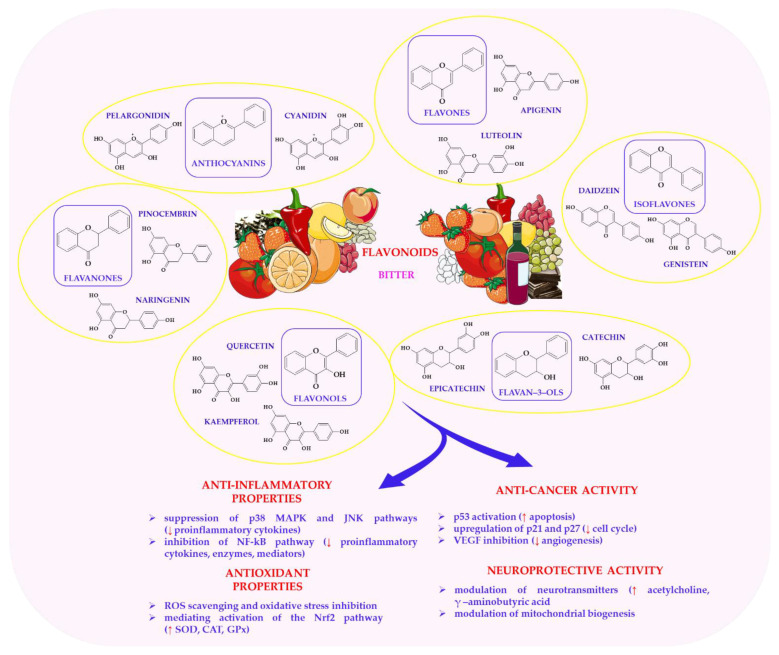
Molecular mechanisms of flavonoid action. Abbreviations: CAT—Catalase; GPx—Glutathione peroxidase; JNK—Janus kinase; p38 MAPK—p38 mitogen-activated protein kinase; NF-кB—Nuclear factor kappa-light-chain-enhancer of activated B cells; Nfr2—Nuclear factor erythroid 2-like 2; ROS—Reactive oxygen species; SOD—Superoxide dismutase; VEGF—Vascular endothelial growth factor. The red arrows indicate a decrease (↓) or increase (↑) in the respective parameter. Image provided by Servier Medical Art (https://smart.servier.com/), licensed under CC BY 4.0 (https://creativecommons.org/licenses/by/4.0/, accessed on 1 May 2025).

The neuroprotective mechanisms of flavonoids involve the inhibition of acetylcholinesterase (AChE) to increase acetylcholine levels and improve cognitive performance [239,240]. They also modulate GABAergic neurotransmission; for example, compounds such as luteolin act as positive modulators of GABA_A receptors, contributing to anxiolytic and sedative effects [241,242]. These pleiotropic actions suggest that flavonoids should be further explored as nutraceutical agents that support mental health and cognitive longevity, particularly in ageing populations. Molecular mechanisms of flavonoid action are shown in Figure 6. This figure shows that flavonoids participate in multiple cellular processes, including antioxidant defence, modulation of enzyme activity and regulation of cell signalling pathways involved in inflammation, apoptosis and metabolism. At the molecular level, flavonoids can scavenge ROS and interact with key proteins and receptors, thereby influencing gene expression and cellular homeostasis. Flavonoids have been associated with reduced risk of chronic diseases such as neurodegeneration and certain cancers, largely due to their anti-inflammatory, antioxidant, and anti-proliferative effects.

Emerging research demonstrates that flavonoids influence the composition of gut microbiota, promoting the growth of beneficial bacteria and enhancing the bioavailability of other phytochemicals [243,244]. This bidirectional interaction, whereby the microbiota also metabolise flavonoids into more bioactive derivatives, represents a critical yet under-explored mechanism in human nutrition that should be incorporated into future clinical and metabolomic studies.

Beyond their biological functions, flavonoids significantly impact the flavour, colour and texture of plant-based foods [135,216]. For example, quercetin and kaempferol impart bitterness and astringency to foods such as apples, onions and tea by interacting with T2R bitter receptors [18,227]. Anthocyanins contribute to vivid red and purple hues, while catechins and epicatechins influence the complex sensory balance of cocoa and wine [245,246]. Flavonoids also interact synergistically with volatile organic compounds—terpenes, aldehydes, and esters—that determine aroma and taste complexity [20,247]. For example, eugenol and vanillin enhance clove-like and sweet vanilla notes [180,248]. The integration of sensory and biochemical data could therefore provide valuable insights for designing functional foods that balance palatability with bioactive potential.

In summary, flavonoids such as quercetin, kaempferol and catechins are multifunctional dietary constituents that link the sensory appeal of plant-based foods with their biological efficacy. Through antioxidant, anti-inflammatory, anticancer and neuroprotective mechanisms, they promote human health and prevent disease [135,192,249]. However, a major scientific challenge lies in bridging the gap between mechanistic insights and real-world bioefficacy, particularly with regard to improving flavonoid stability, bioavailability, and matrix interactions. Future research should focus on integrative approaches combining metabolomics, gut microbiome profiling, and sensory science to better understand how flavonoids function within complex dietary systems. This holistic approach will inform evidence-based strategies for utilising flavonoids as integral components of health-promoting, sensorially appealing plant-based diets.

## 7. Phytochemicals: Linking Flavour Development, Antioxidant and Anti-Inflammatory Effects in Plant-Based Foods

These compounds play a central role in shaping sensory attributes and health-promoting properties [5,218]. They influence flavour development, aroma and mouthfeel by interacting with taste receptors, olfactory pathways and metabolic processes. At the same time, they modulate the molecular signalling pathways that underpin antioxidant and anti-inflammatory responses [215,250].

Table 1 summarises the main bioactive compounds in plant-based foods, their sensory characteristics, bioactive effects and plant sources.

Flavonoids such as quercetin and kaempferol modulate the nuclear factor kappa B (NF-κB) pathway, which is a key regulator of inflammation and immune responses [258]. NF-κB activation induces the production of pro-inflammatory cytokines, which can indirectly affect flavour development by altering the metabolic pathways responsible for synthesising volatile aroma compounds [259]. Furthermore, NF-κB signalling influences the composition of the gut microbiota, thereby affecting sensory perception through microbial fermentation and the production of metabolites that interact with food components [260,261]. Inhibiting NF-κB activation with flavonoids and phenolic compounds can therefore attenuate chronic inflammation and reduce the risk of diseases such as arthritis, cardiovascular disease and diabetes, while also improving sensory quality [191,192,262,263,264].

Another critical pathway influenced by phytochemicals is the nuclear factor erythroid 2-related factor 2 (Nrf2)/antioxidant response element (ARE) system, which controls cellular antioxidant defences [228,265]. Compounds such as curcumin, resveratrol, sulforaphane, quercetin and baicalein can activate Nrf2 by modifying cysteine residues on Kelch-like ECH-associated protein 1 (Keap1). This leads to increased transcription of antioxidant and phase II detoxifying enzymes, including superoxide dismutase (SOD), glutathione peroxidase (GPx) and catalase (CAT) [266,267,268,269,270]. These enzymes scavenge reactive oxygen species (ROS), protecting cellular components from oxidative damage and maintaining the integrity of flavour-active phytochemicals, such as polyphenols and carotenoids, which are susceptible to oxidative degradation during food processing and storage [30,271]. Activation of Nrf2 also supports tissue repair and cellular homeostasis, as well as protecting against diseases related to oxidative stress, including cardiovascular and neurodegenerative disorders, and certain cancers [272,273,274,275].

Phytochemicals also modulate the mitogen-activated protein kinase (MAPK) and activator protein 1 (AP-1) pathways, thereby contributing to anti-inflammatory responses and cellular homeostasis [15,250,276]. Through these mechanisms, they stabilise cellular environments, reduce tissue damage caused by oxidative and inflammatory stress, and preserve the sensory and functional quality of plant-based foods.

These molecular mechanisms not only have physiological effects, but also influence the functional stability and shelf life of plant-based products. Antioxidant and anti-inflammatory activities mitigate oxidative deterioration and rancidity, thereby maintaining the integrity of lipids and other sensitive food components [30,131,277]. Consequently, foods rich in phytochemicals offer dual benefits: enhanced sensory appeal and improved nutritional and protective properties. This aligns with the growing consumer demand for functional foods that support long-term health [278,279,280,281].

In conclusion, the modulation of the NF-κB, Nrf2, MAPK and AP-1 pathways by phytochemicals provides a mechanistic link between flavour development, antioxidant defence and anti-inflammatory effects. By influencing sensory perception and cellular health, these compounds improve the quality, stability and functional properties of plant-based foods. A deeper understanding of these molecular interactions is essential for optimising food formulations and processing strategies, as well as the health-promoting potential of plant-based diets [12,15,221,233,250].

## 8. Optimising Phytochemical Stability and Bioavailability in Plant-Based Foods Through Processing

The processing of plant foods plays a crucial role in modulating the stability and bioavailability of phytochemicals, which, in turn, influences both the sensory qualities and functional health benefits of these foods [282,283]. Various food processing techniques, including thermal treatments, pH adjustments, and oxidative conditions, can significantly affect the integrity of phytochemicals, altering their chemical structure and bioactivity [30,284]. These changes are particularly important in understanding how processing affects the flavour, nutritional value, and overall health-promoting properties of plant-based diets.

One of the most common processing methods that affect phytochemical stability is thermal processing. High temperatures can lead to the degradation of sensitive phytochemicals such as flavonoids and terpenes, which are critical to the flavour and functional benefits of plant-based foods [283,285]. Flavonoids, for example, are known to undergo structural changes under heat, potentially breaking down into smaller, more bioavailable metabolites [286]. However, this degradation can alter their original bioactivity [287]. In contrast, terpenes, which contribute significantly to the aromatic intensity of plant foods, are volatile compounds that are highly susceptible to heat-induced loss. The evaporation of these compounds during the cooking process can reduce the aromatic complexity of foods, thereby reducing their sensory appeal [20]. In addition, oxidative conditions commonly encountered during cooking or food storage can lead to the formation of reactive species that further degrade phytochemicals, thereby compromising their potential health benefits [288].

To counteract the negative effects of heat and oxidative degradation, innovative technologies such as encapsulation and nanoformulation have been proposed to preserve the stability and bioavailability of sensitive phytochemicals [289,290]. These methods involve encapsulating volatile compounds, such as terpenes and phenolic acids, in protective carriers to protect them from environmental stressors during food preparation and storage [290,291]. Nanoformulation techniques also enhance the bioavailability of phytochemicals by improving their solubility and facilitating their absorption in the gastrointestinal tract [292]. These advanced technologies can maximise the stability and functional benefits of phytochemicals, ensuring that plant-based foods retain their nutritional and sensory properties despite processing [289,290].

While certain food processing methods can degrade phytochemicals, fermentation has been shown to offer distinct advantages by increasing the bioavailability of certain bioactive compounds [293]. During fermentation, microorganisms such as bacteria and yeasts can modify the chemical structure of phytochemicals, making them more bioavailable for absorption by the human body [294,295]. For example, microbial fermentation has been shown to increase the concentration of bioactive flavonoid metabolites, which not only enhance the flavour complexity of plant-based foods, but also improve their potential health benefits [296]. This process can also lead to the production of beneficial metabolites such as short-chain fatty acids (SCFAs), which support gut health and influence the flavour profile of fermented foods [297,298]. In addition, fermentation can reduce levels of anti-nutritional factors such as phytates and oxalates, thereby improving the bioavailability of minerals and other nutrients in plant-based foods [299].

In addition to thermal and microbial treatments, pH adjustments during food processing can affect the stability and bioavailability of phytochemicals [284]. Acidic or alkaline conditions can affect the solubility, stability, and ionisation of phytochemicals, thereby influencing their sensory attributes and bioactivity [300]. For example, variations in pH can alter the colour, flavour and aroma of flavonoids and other bioactive compounds, affecting both consumer acceptance and functional health effects [301]. It is essential to optimise pH conditions during food processing to preserve the desirable properties of phytochemicals.

To maximise the benefits of phytochemical-rich diets, it is essential to understand the biochemical transformations that occur during food processing. Knowledge of how different processing techniques affect the stability, bioavailability, and functionality of phytochemicals can inform food formulation strategies that preserve both sensory integrity and health benefits [284]. By combining appropriate processing methods, such as controlled thermal processing, fermentation, and innovative encapsulation technologies, food manufacturers can ensure that plant-based foods retain their full range of beneficial compounds. This helps to maximise their nutritional value, flavour complexity, and health-promoting effects, thereby encouraging the consumption of plant-based foods as part of a healthy, sustainable diet.

Table 2 summarises the main bioactive compounds found in plant-based foods, their sensory characteristics, bioactive effects, and plant sources.

In summary, food processing significantly affects the stability and bioavailability of phytochemicals, which are key determinants of both the flavour and functional properties of plant-based foods. While some processes, such as thermal treatment and oxidative exposure, can degrade phytochemicals, advanced technologies such as encapsulation and fermentation can enhance their stability and bioavailability. By understanding and optimising the biochemical transformations that occur during processing, it is possible to preserve the desirable properties of phytochemicals and maximise their health benefits, ultimately supporting the promotion of plant-based diets.

## 9. Study Limitations

This review summarises the current knowledge on the sensory and health-promoting properties of phytochemicals in plant-based foods. However, several limitations should be acknowledged. Firstly, despite efforts to include a wide range of studies from multiple databases, publication bias and the exclusion of non-English literature may have limited the scope of the review. Secondly, the diversity of experimental models, analytical methods and food matrices across the studies makes direct comparisons difficult and may affect the generalisability of the conclusions. Thirdly, while mechanistic insights are highlighted, many studies focus on in vitro or animal models and human clinical data on the combined effects of multiple phytochemicals on flavour perception and health outcomes remains limited.

Finally, the impact of food processing on phytochemical stability and bioavailability is complex and multifaceted. For example, thermal treatments such as cooking or pasteurisation can enhance or degrade bioactive compounds. Moderate heating can increase the extractability of polyphenols or carotenoids by breaking down plant cell walls, whereas prolonged heating can lead to their degradation. Fermentation can enhance bioavailability and generate new bioactive metabolites, often improving health-promoting properties and sensory profiles by reducing bitterness or astringency, for example. Encapsulation techniques have been shown to protect sensitive compounds, such as carotenoids and volatile terpenes, from oxidation and volatilisation, thereby maintaining functional properties and aroma. Other methods, including pH adjustment, high-pressure processing and light protection, can also influence phytochemical stability and the perception of taste and colour; however, their effects depend heavily on the specific food matrix and processing parameters.

Overall, although general trends can be identified, the effects of processing are highly context-dependent. This makes it challenging to translate laboratory findings directly into real-world dietary applications. Recognising these limitations highlights the need for further controlled human studies and standardised methodologies. This will strengthen the evidence base and guide the development of functional, plant-based foods with optimised sensory and health-promoting properties.

## 10. Conclusions

This review examines the integral role of phytochemicals in shaping the sensory characteristics and health-promoting properties of plant-based foods. By analysing the biochemical pathways underlying the biosynthesis of key classes of phytochemicals, including flavonoids, terpenes, and phenolic compounds, the review elucidates their significant contributions to the sensory perception and the overall aromatic complexity of plant-based foods. These compounds not only define the sensory profile but also influence critical molecular mechanisms that are central to mediating the health benefits associated with plant-based diets.

In addition to their sensory properties, phytochemicals are involved in the modulation of key signalling pathways, such as NF-κB, Nrf2, MAPK, and AP-1, which regulate inflammation, oxidative stress, and cell survival. These pathways are critical for the antioxidant and anti-inflammatory effects of phytochemicals, highlighting their potential to reduce the risk of chronic diseases, such as cardiovascular disease, cancer, and neurodegenerative disorders. The intricate interplay between these molecular mechanisms reinforces the importance of phytochemicals in promoting human health and emphasises their dual function as sensory enhancers and health modulators.

In addition, the review emphasises the impact of food processing on the stability and bioavailability of phytochemicals, which is crucial to ensuring that the functional properties of these compounds are maintained throughout food preparation and storage. The discussion highlights the need for optimised processing techniques that minimise degradation while enhancing the bioavailability of phytochemicals. The application of innovative technologies, such as nanoencapsulation, fermentation, and controlled thermal processing, is examined as a promising strategy to improve the retention of phytochemical bioactivity, thereby ensuring the preservation of both their flavour and functional efficacy.

## Figures and Tables

**Table 1:** Summary of main bioactive compounds in plant-based foods, their sensory characteristics, bioactive effects and plant sources.

Compound	Plant Source	Sensory Characteristics	Bioactive Effect	References
Quercetin	Apples, onions, tea, berries	Bitter, astringent	Antioxidant, anti-inflammatory, anticancer, neuroprotective, anti-diabetic	[135,220,227,251]
Kaempferol	Kale, spinach, tea	Slightly bitter	Antioxidant, anti-inflammatory, neuroprotective, anticancer	[220,252,253]
Catechins (e.g., epicatechin)	Green tea, dark chocolate	Astringent, slightly bitter	Antioxidant, cardioprotective, neuroprotective, anticancer	[218,223]
Anthocyanins	Berries, hibiscus	Tart, fruity	Antioxidant, anti-inflammatory, neuroprotective	[135,254]
Apigenin	Chamomile	Sweet, floral	Neuroprotective, anti-inflammatory, antioxidant	[228,255]
Resveratrol	Red wine, grapes	Slightly bitter, fruity	Antioxidant, anti-inflammatory, cardioprotective, anticancer	[245,256]
Pinocembrin	Buckwheat honey	Slightly bitter, robust	Antioxidant, neuroprotective	[257]
Limonene	Citrus fruits	Citrus, fresh	Antioxidant, anti-inflammatory, antimicrobial	[20,154]
Pinene	Pine needles, rosemary	Pine, fresh, resinous	Anti-inflammatory, antimicrobial, antioxidant	[20,160]
Myrcene	Hops, mango, lemongrass	Earthy, herbaceous, tropical	Anti-inflammatory, analgesic	[20,156]
Linalool	Lavender, mint	Floral, sweet	Sedative, anti-anxiety, antioxidant	[20,161]
Terpinene	Thyme, oregano	Citrus, herbaceous	Antioxidant, antimicrobial	[20,162]
Caryophyllene	Black pepper, cloves	Spicy, peppery	Anti-inflammatory, analgesic, antimicrobial	[20,163]
Geraniol	Geranium, rose	Sweet, floral	Antioxidant, anti-inflammatory	[20,164]
Citronellol	Rose, geranium	Sweet, floral	Antioxidant, antimicrobial	[20,164]
Eugenol	Cloves	Spicy, clove-like	Antimicrobial, antioxidant, analgesic	[159,180]
Vanillin	Vanilla pods	Sweet, creamy	Antioxidant, antimicrobial	[20,248]
Chlorogenic acid	Coffee, apples	Slightly bitter, earthy	Antioxidant, anti-diabetic	[179,193]
Ellagic acid	Berries	Tart, earthy	Antioxidant, anticancer	[176,177]
Gallic acid	Dark honey, dark chocolate	Astringent, slightly bitter	Antioxidant, anticancer	[182,213]

**Table 2:** The effects of processing techniques on the stability, bioavailability and sensory properties of major plant-derived bioactive compounds.

Major Compounds	Plant Sources	Processing Technique	Effect on Stability	Effect on Bioavailability	Effect on Sensory Profile	Key References
Quercetin, kaempferol, catechins, anthocyanins	Apples, onions, tea, red wine, dark chocolate	Thermal (blanching, pasteurization, cooking)	Partial degradation; anthocyanins sensitive; catechins moderately stable	Slight increase due to breakdown into smaller metabolites, but bioactivity may change	Color fading; slight bitterness remains	[283,286,287]
Quercetin, kaempferol	Berries, citrus	Fermentation	Generally stable; some microbial transformation	Enhanced due to conversion of glycosides to aglycones	Improved flavor complexity	[293,296]
Quercetin, catechins	Dark chocolate, tea	Encapsulation/Nanoformulation	Stabilized against heat, light, and oxidation	Significantly improved solubility and absorption	Minimal impact on taste; reduced astringency	[289,290,292]
Quercetin, kaempferol	Berries, Citrus	pH adjustment	Acidic or alkaline conditions can alter stability	Changes in solubility and ionization may enhance or reduce bioavailability	Altered color, flavor, aroma	[300,301]
Limonene, pinene, myrcene, linalool	Citrus, herbs, mango, hops	Thermal	Highly volatile; significant losses	Reduced due to degradation	Loss of aroma and aromatic intensity	[20,285]
Linalool, geraniol	Lavender, mint, rose	Fermentation	Slight biotransformation; relatively stable	Slightly improved due to release from matrix	Enhanced floral notes; new aroma compounds	[164,294]
Limonene, pinene	Citrus, pine	Encapsulation/Nanoformulation	High stability; protected from heat and oxidation	Improved controlled release and absorption	Preserved aroma; prolonged sensory perception	[290,291]
Chlorogenic acid, ellagic acid, tannins, resveratrol	Coffee, berries, tea, red wine	Thermal	Partial degradation; tannins moderately stable	Extractability may increase but degradation reduces bioactivity	Bitterness and astringency may increase	[187,288]
Catechins, flavonoids	Tea, coffee, cocoa	Fermentation	Microbial transformation can produce more bioactive metabolites	Increased bioavailability	Enhanced flavor complexity; production of SCFAs	[193,298]
Flavonoids, tannins	Berries, chocolate	Encapsulation/Nanoformulation	Protected from oxidation	Increased absorption; targeted release	Minimal sensory change	[125,184]
Flavonoids, phenolic acids	Various fruits and vegetables	Oxidative stress/Storage conditions	Degradation due to reactive oxygen species	Reduced if not stabilized	Color and flavor changes	[30,288]

## Data Availability

No new data were created or analysed in this study. Data sharing is not applicable to this article.
